# Clinical Characteristics and Serum Vitamin D Levels in Patients with MRONJ Associated with Antiresorptive Therapy: A Retrospective Study

**DOI:** 10.3390/medicina62071394

**Published:** 2026-07-18

**Authors:** Ivona Mihaela Hum, Laura Atyim, Raluca Maracineanu, Robert Folescu, Loredana Gabriela Stana, Roxana Folescu

**Affiliations:** 1 Doctoral School, “Victor Babeș” University of Medicine and Pharmacy, 300041 Timisoara, Romania; ivona.ursu@umft.ro (I.M.H.); raluca.zibileanu@umft.ro (R.M.); 2 Department of Balneology, Medical Recovery and Rheumatology, Family Medicine University Clinic, Center for Preventive Medicine, Center for Advanced Research in Cardiovascular Pathology and Hemostaseology, “Victor Babeș” University of Medicine and Pharmacy, 300041 Timisoara, Romania; folescu.roxana@umft.ro; 3 Faculty of Medicine, “Victor Babes” University of Medicine and Pharmacy, 300041 Timisoara, Romania; robert.folescu@student.umft.ro; 4 Department I, Discipline of Anatomy and Embryology, “Victor Babes” University of Medicine and Pharmacy, 300041 Timisoara, Romania; loredana.stana@umft.ro

**Keywords:** medication-related osteonecrosis of the jaw (MRONJ), vitamin D, bone resorption inhibitors

## Abstract

*Background and Objectives*: Medication-related osteonecrosis of the jaw (MRONJ) is a severe complication associated with antiresorptive therapy. Although several local and systemic risk factors have been documented, the role of metabolic biomarkers and their relationship with clinical characteristics remains insufficiently investigated. The aim of the present study was to evaluate the relationships between antiresorptive therapy, serum vitamin D levels, demographic characteristics, and lesion location in patients diagnosed with MRONJ. *Materials and Methods*: This retrospective study included 72 patients diagnosed with MRONJ, divided into three groups according to the antiresorptive therapy administered: zoledronic acid (*n* = 30), denosumab (*n* = 22), and oral bisphosphonates (*n* = 20). Clinical and demographic data, including age, sex, smoking status, serum vitamin D levels, number of extracted teeth, lesion location (mandibular versus maxillary), and associated comorbidities, were obtained from medical records. Statistical analysis was performed using Welch’s ANOVA, Welch’s *t*-test, chi-square, Fisher’s exact, and Pearson and Spearman correlation analyses. *Results*: No significant differences in serum vitamin D levels were observed among the three antiresorptive therapy groups. However, patients with mandibular MRONJ had significantly lower serum vitamin D levels than those with maxillary involvement (*p* = 0.046). Lower serum vitamin D levels were independently associated with mandibular MRONJ after multivariable adjustment. Female sex and thyroid disease were more frequent in the oral bisphosphonate group. Additionally, patient age was moderately positively correlated with the number of extracted teeth (*r* = 0.440, *p* < 0.001). *Conclusions*: This retrospective study identified a possible association between lower serum vitamin D levels and mandibular MRONJ. Given the observational design of the study, these findings should be interpreted with caution and require confirmation in larger prospective studies.

## 1. Introduction

Medication-related osteonecrosis of the jaw (MRONJ) is an adverse reaction associated with antiresorptive or antiangiogenic medications that significantly impairs patients’ quality of life by causing infections, chronic pain, and eating and speech disorders [1,2]. According to the American Association of Oral and Maxillofacial Surgeons (AAOMS), the diagnosis of MRONJ is established based on the following criteria: (i) exposed bone or a fistula in the maxillofacial region for more than 8 weeks; (ii) current or previous antiresorptive or antiangiogenic treatment; and (iii) absence of a history of radiotherapy to the jaws [3].

MRONJ is most commonly associated with the administration of bisphosphonates and denosumab, drugs widely prescribed in oncological conditions, including bone metastases in breast or prostate cancer or multiple myeloma, as well as for non-oncological pathologies, such as osteoporosis and Paget’s disease [3,4].

Since the first cases of MRONJ were reported in 2003 [5], awareness of the severity of this pathology has increased significantly; however, the exact underlying pathophysiological mechanisms remain multifactorial and incompletely understood. Current hypotheses involve an interplay between bone remodeling inhibition, impaired angiogenesis, local trauma, infection, and persistent inflammation [3,6,7,8]. The severity and clinical presentation of the disease vary considerably depending on the primary condition, as well as the specific antiresorptive agent, cumulative dose, and route of administration [9,10]. In addition, several local and systemic risk factors can influence the development of MRONJ, including lesion location, oncological pathology, corticosteroid therapy, and smoking [3].

Although local factors, such as trauma due to dental extractions, are well-known triggers of MRONJ [11], contemporary maxillofacial surgery increasingly emphasizes precision diagnostics to identify systemic biomarkers that affect bone healing. Within this context, vitamin D (25-hydroxyvitamin D) has emerged as a crucial metabolic factor, playing multiple roles in calcium-phosphate homeostasis, bone turnover, and immune modulation [12]. Vitamin D deficiency has been progressively linked to an elevated risk of MRONJ development and severity, with suboptimal serum levels frequently reported in patients receiving antiresorptive therapies, potentially compounding impaired bone healing [13,14,15]. However, the specific interplay among serum vitamin D status, the anatomical location of MRONJ lesions, and the type of antiresorptive medication remains underexplored in current clinical research.

The primary aim of this retrospective study was to investigate the association between serum vitamin D levels and the clinical characteristics of patients with MRONJ receiving antiresorptive therapy. Secondary analyses evaluated associations between serum vitamin D levels, demographic characteristics, lesion location, and the number of extracted teeth.

## 2. Materials and Methods

### 2.1. Study Design and Patient Selection

**Figure 1:**
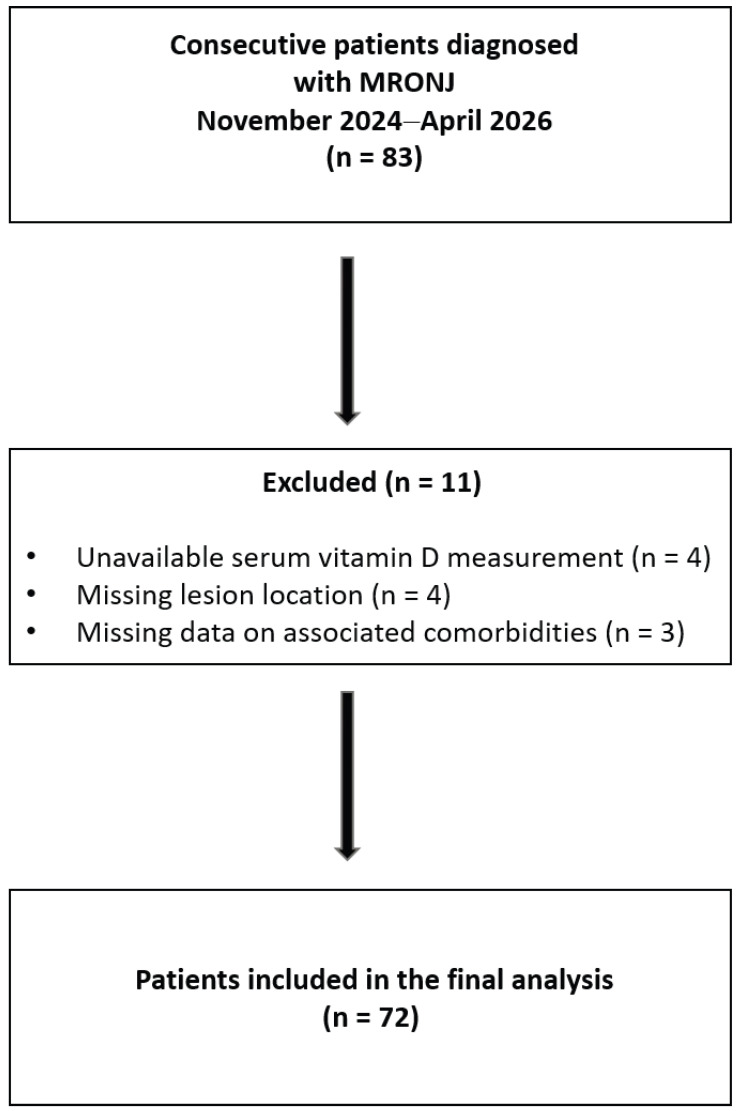
Flow diagram of the patient selection process.

A retrospective observational study was conducted during an 18-month period (November 2024–April 2026) at the Department of Oral and Maxillofacial Surgery, Municipal Emergency Hospital, affiliated with the Victor Babeș University of Medicine and Pharmacy, Timișoara. A total of 83 consecutive patients diagnosed with MRONJ during the study period were screened for eligibility. Patients were excluded if serum 25-hydroxyvitamin D measurements obtained at hospital admission were unavailable (*n* = 4), lesion location was not documented (*n* = 4), or information regarding associated comorbidities was incomplete (*n* = 3). Consequently, 72 patients met the eligibility criteria and were included in the final analysis (Figure 1).

This study is reported in accordance with the Strengthening the Reporting of Observational Studies in Epidemiology (STROBE) Statement.

The study was conducted in accordance with the principles of the Declaration of Helsinki and was approved by the Ethics Committee of the Victor Babeș University of Medicine and Pharmacy, Timișoara, Romania (No 106/11 October 2024).

The diagnosis of MRONJ was previously established in all cases according to the AAOMS criteria [3], requiring the presence of an oral fistula or persistent gingival dehiscence with underlying bone exposure for more than 8 weeks. Serum 25-hydroxyvitamin levels were measured at the time of hospital admission, prior to any surgical or pharmacological intervention, using an electrochemiluminescence immunoassay. The month of blood sampling was also recorded to account for potential seasonal variation in vitamin D status. According to the reference ranges used by our laboratory, serum vitamin D levels < 30 µg/L (including deficient and insufficient levels) were considered suboptimal, whereas levels ≥ 30 µg/L were considered within the normal range.

### 2.2. Data Collection

Clinical and demographic data were retrospectively extracted from the patients’ medical records. The analyzed variables included age, sex, area of residence, smoking status, serum vitamin D levels, month of blood sampling, number of extracted teeth, lesion location (mandibular or maxillary), diabetes mellitus, cardiovascular disease, thyroid disease, type of antiresorptive treatment, and the underlying diagnosis, when applicable.

Additional concomitant medications, including antihypertensive, anticoagulant, antidiabetic, corticosteroid, and thyroid-related medications, were also recorded.

Serum vitamin D levels were expressed in µg/L.

### 2.3. Antiresorptive Therapy Groups

Patients were divided into three groups according to the administered antiresorptive therapy: (1) zoledronic acid, (2) denosumab, and (3) oral bisphosphonates.

Oral bisphosphonates were administered exclusively to patients with osteoporosis, whereas zoledronic acid and denosumab were predominantly used for oncologic indications and administered intravenously and subcutaneously, respectively.

### 2.4. Statistical Analysis

Statistical analysis was performed using JASP software (version 0.18.3; JASP Team, University of Amsterdam, Amsterdam, The Netherlands; https://jasp-stats.org).

The normality of continuous variables was assessed using the Shapiro–Wilk test. Continuous variables were expressed as mean ± standard deviation (SD), whereas categorical variables were reported as absolute numbers and percentages (*n*, %).

Differences among the three therapeutic groups for continuous variables were evaluated using Welch’s ANOVA. When the overall test was significant, Games–Howell post hoc comparisons were performed. Comparisons between two groups (including serum vitamin D levels, body mass index, and duration of antiresorptive therapy according to MRONJ anatomical location) were performed using Welch’s *t*-test. Categorical variables were analyzed using the chi-square test (χ^2^) or Fisher’s exact test when expected cell counts were below 5. Correlations between continuous variables, including serum vitamin D levels, age, number of extracted teeth, and duration of antiresorptive therapy, were assessed using Pearson’s (*r*) and Spearman’s (*ρ*) correlation coefficients.

To evaluate the potential influence of seasonal variation on serum vitamin D levels, patients were categorized according to the season of blood sampling (spring, summer, autumn, and winter). Serum 25-hydroxyvitamin D concentrations were compared among seasons using one-way ANOVA, followed by Tukey’s post hoc test for pairwise comparisons. A multivariable binary logistic regression analysis was additionally performed to evaluate whether serum vitamin D levels were independently associated with MRONJ anatomical location after adjustment for age, sex, diabetes mellitus, and antiresorptive therapy.

Statistical significance was set at *p* < 0.05.

## 3. Results

The study evaluated 72 patients diagnosed with MRONJ, stratified into three distinct therapeutic groups based on the administered antiresorptive agent: zoledronic acid (*n* = 30), denosumab (*n* = 22), and oral bisphosphonates (*n* = 20). Notably, oral bisphosphonates were prescribed exclusively for osteoporotic patients. In contrast, zoledronic acid (intravenous) and denosumab (subcutaneous) were predominantly utilized for oncologic indications. Within the oncologic cohort, breast cancer represented the most frequent primary malignancy (*n* = 25), followed by prostate cancer (*n* = 16), multiple myeloma (*n* = 5), and bronchopulmonary cancer (*n* = 4). Isolated cases of renal and brain malignancies were also recorded. Baseline demographic and clinical characteristics across the three therapeutic groups are summarized in Table 1.

Comparative analysis revealed no statistically significant differences among the therapeutic groups regarding age (*p* = 0.157), smoking status (*p* = 0.664), serum vitamin D levels (*p* = 0.110), number of extracted teeth (*p* = 0.324), MRONJ lesion location (*p* = 0.385), diabetes mellitus (*p* = 0.753), or cardiovascular disease (p = 0.668). Female sex was significantly associated with oral bisphosphonate therapy (100%) compared to the zoledronic acid and denosumab regimens (*p* = 0.003). Similarly, thyroid disease demonstrated a significantly higher prevalence in the oral bisphosphonate group than in the other therapeutic cohorts (*p* = 0.005). The duration of antiresorptive therapy differed significantly among the three treatment groups (Welch’s ANOVA, *F* = 86.82, *p* < 0.001). Patients receiving oral bisphosphonates had the longest treatment duration (42.5 ± 12.3 months), followed by denosumab (13.7 ± 2.2 months) and zoledronic acid (9.0 ± 2.5 months). Games–Howell post hoc analysis demonstrated significant differences between all pairwise comparisons (all *p* < 0.001). The distribution of AAOMS stages did not differ significantly among the three antiresorptive treatment groups (*p* = 0.988).

Concomitant medications according to antiresorptive treatment groups are summarized in Table 2. Their distribution was generally comparable across groups and reflected the differences in underlying comorbidities.

Correlation analyses evaluated the associations between age, serum vitamin D levels, and the number of extracted teeth (Table 3).

A moderate positive correlation was observed between patient age and the number of extracted teeth (Pearson *r* = 0.440, *p* < 0.001; Spearman *ρ* = 0.395, *p* < 0.001), indicating a greater history of dental interventions or extractions in older individuals. Conversely, no statistically significant correlations were identified between serum vitamin D levels and either age or the number of extracted teeth.

No significant correlation was observed between serum vitamin D levels and the duration of antiresorptive therapy (Pearson *r* = − 0.223, *p* = 0.060; Spearman *ρ* = − 1.112, *p* = 0.350).

Clinical and demographic parameters were further compared based on the specific anatomical location of the MRONJ lesions (mandibular versus maxillary) (Table 4).

**Figure 2:**
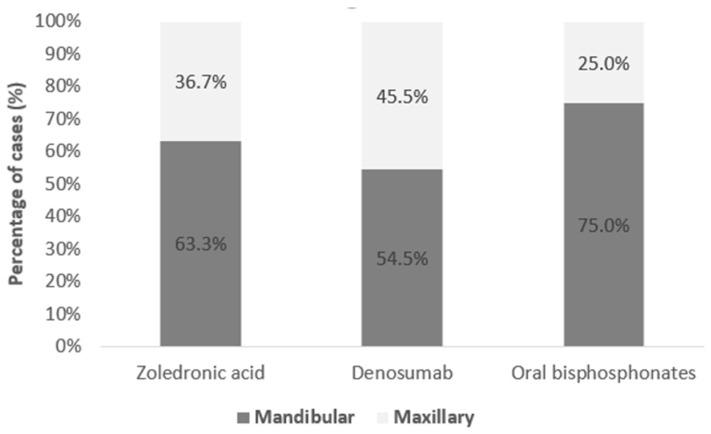
Percentage distribution of mandibular and maxillary MRONJ lesions according to antiresorptive therapy.

Mandibular involvement was more prevalent overall, accounting for 63.9% of all cases, and remained the predominant site across all therapeutic regimens, particularly in patients treated with zoledronic acid and oral bisphosphonates (Figure 2).

**Figure 3:**
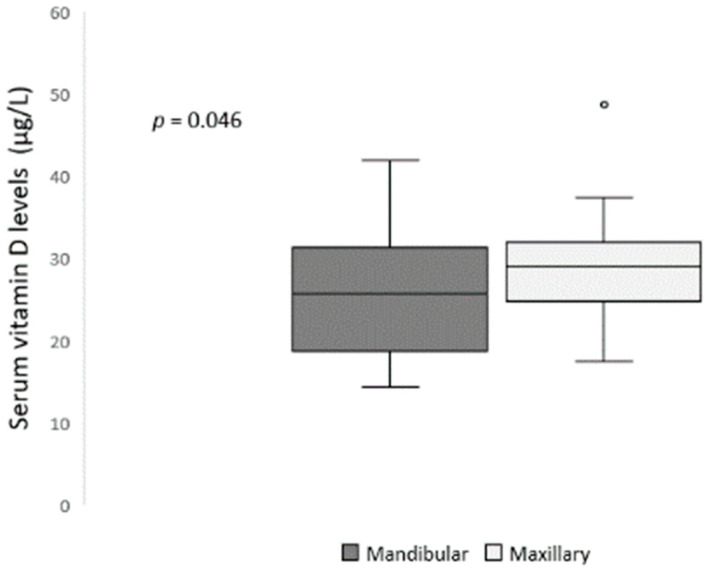
Boxplot representation of serum vitamin D levels according to MRONJ anatomical location (mandibular vs. maxillary).

Patients with mandibular MRONJ had significantly lower mean serum vitamin D levels than those with maxillary involvement (25.4 ± 6.6 vs. 28.7 ± 6.5 μg/L, *p* = 0.046), as illustrated in Figure 3. However, when serum vitamin D levels were categorized as suboptimal (<30 μg/L) or normal (≥30 μg/L), no significant difference in the prevalence of suboptimal vitamin D status was observed between mandibular and maxillary lesions (71.7% vs. 53.8%, *p* = 0.126).

To evaluate the potential influence of seasonality, an additional analysis according to the season of blood sampling was performed. Serum 25-hydroxyvitamin D levels differed significantly among seasons (one-way ANOVA, *F* = 3.719, *p* = 0.015). Mean vitamin D concentrations were highest during summer (31.64 ± 6.35 μg/L) and lowest during winter (23.95 ± 4.51 μg/L). Tukey’s post hoc analysis demonstrated a significant difference between summer and winter (*p* = 0.008), whereas no other pairwise comparisons reached statistical significance.

Body mass index was comparable between patients with mandibular and maxillary MRONJ (24.68 ± 3.11 vs. 24.97 ± 3.11 kg/m^2^, *p* = 0.705). Likewise, the duration of antiresorptive therapy was similar between the two groups (20.5 ± 15.7 vs. 18.4 ± 16.3 months, *p* = 0.594). The distribution of AAOMS stages was also comparable between mandibular and maxillary MRONJ lesions (*p* = 0.686). No other clinical or demographic factors assessed, including age, smoking status, diabetes, gender, or antiresorptive class, demonstrated a significant association with the anatomical location of the lesion. To determine whether serum vitamin D levels were independently associated with MRONJ anatomical location, a multivariable binary logistic regression analysis was performed including serum vitamin D levels, age, sex, diabetes mellitus, and antiresorptive therapy. The overall model was statistically significant (χ^2^ = 12.78, *p* = 0.047). Higher serum vitamin D levels were independently associated with lower odds of mandibular MRONJ (OR = 0.912, 95% CI 0.834–0.998, *p* = 0.044). Likewise, increasing age was independently associated with lower odds of mandibular involvement (OR = 0.928, 95% CI 0.867–0.992, *p* = 0.028). Sex, diabetes mellitus, and antiresorptive therapy were not significant predictors of MRONJ anatomical location (Table 5).

## 4. Discussion

MRONJ remains one of the most serious complications associated with antiresorptive medication in oncologic and osteoporotic patients undergoing long-term treatment [3,16]. Despite the growing number of studies published in recent years, the pathophysiological mechanisms underlying MRONJ remain incompletely understood and are considered multifactorial. This retrospective study evaluated the clinical and demographic characteristics of patients diagnosed with MRONJ according to the antiresorptive therapy administered, with particular emphasis on serum vitamin D levels and lesion location.

The main results of the study were: (i) no significant differences between the therapeutic groups in terms of age, smoking status, serum vitamin D levels and number of extracted teeth; (ii) a significantly higher prevalence of female sex and thyroid pathology in the group treated with oral bisphosphonates; (iii) a moderate positive correlation between age and number of extracted teeth; (iv) a predominant mandibular involvement compared to maxillary location and (v) significantly lower vitamin D levels in patients with mandibular lesions compared to those with maxillary involvement.

Bisphosphonates and denosumab are the antiresorptive agents most frequently implicated in the development of MRONJ, especially in high-dose therapy [3,17,18]. The absence of statistically significant differences among the therapeutic groups may be due to similar clinical outcomes across these therapies. Although intravenous bisphosphonates, such as zoledronic acid, accumulate in the bone matrix for prolonged periods, while denosumab acts through inhibition of the RANKL pathway and has a shorter half-life, both therapeutic agents suppress osteoclast-mediated bone resorption and may contribute to MRONJ development in the presence of local risk factors [19,20].

The comparison between oral and parenteral antiresorptive agents should be interpreted with caution because these medications are prescribed for different clinical indications. In our cohort, oral bisphosphonates were used exclusively in patients with osteoporosis, whereas intravenous zoledronic acid and denosumab were administered predominantly to oncologic patients. Consequently, the observed differences between treatment groups may partly reflect differences in the underlying diseases rather than the effects of the antiresorptive agents themselves. Given the limited sample size and the strong overlap between treatment indication and underlying diagnosis, further adjustment was unlikely to provide robust estimates. The predominance of female patients in the oral bisphosphonate group is consistent with the epidemiology of osteoporosis, which occurs predominantly in postmenopausal women [21,22]. Similar findings have been reported in other studies, in which female sex predominated in patients treated with oral bisphosphonates, due to the epidemiological characteristics of osteoporosis [23,24].

Furthermore, thyroid pathology was significantly more common in patients treated with oral bisphosphonates. Associated endocrine dysfunctions may influence bone remodeling and tissue repair capacity. Thyroid hormones play an important role in osteoclastic activity and skeletal metabolism, influencing bone turnover in patients with antiresorptive treatment [25]. In addition, altered thyroid hormone levels can indirectly affect bone mineral density and vitamin D metabolism [26].

Vitamin D plays an essential role in bone mineral metabolism and immune response regulation [27]. Several studies have suggested that vitamin D deficiency may contribute to impaired bone healing and increased susceptibility to MRONJ development [9,28,29,30]. In the present study, although serum vitamin D levels did not differ significantly among the three therapeutic groups, patients with mandibular lesions presented significantly lower levels compared with those with maxillary involvement (*p* = 0.046). Notably, when serum vitamin D levels were categorized as suboptimal (<30 μg/L) or normal (≥30 μg/L), the prevalence of suboptimal vitamin D status did not differ significantly between mandibular and maxillary lesions (71.7% vs. 53.8%, *p* = 0.126).

Our findings should also be interpreted in the context of previous studies investigating the role of vitamin D in MRONJ [13,14]. Heim et al. reported lower serum vitamin D levels in patients with MRONJ than in antiresorptive-treated controls without osteonecrosis, suggesting a potential role of vitamin D deficiency in MRONJ development. However, unlike that study, our cohort lacked a matched control group of antiresorptive-treated patients without MRONJ, and information regarding vitamin D supplementation was not consistently available [29]. More recently, Michalak et al. evaluated high-dose vitamin D supplementation in patients with MRONJ and reported that supplementation may reduce the risk of severe disease when administered prophylactically before tooth extraction, whereas no significant effect on postoperative healing was observed once MRONJ had already developed [30].

The lack of significant differences in serum vitamin D concentrations among treatment groups is likely influenced by differences in the underlying clinical populations. Cancer-related factors, concomitant therapies, nutritional status, and systemic inflammation may independently influence serum vitamin D concentrations and obscure treatment-related differences [31,32]. Future studies including more homogeneous oncologic cohorts are warranted.

Seasonal variation should also be considered when interpreting the present results. Serum vitamin D concentrations were significantly higher during summer than during winter, consistent with the known seasonal fluctuation of vitamin D status. Residual confounding related to sun exposure, dietary habits, laboratory methodology, geographic location, and unrecorded vitamin D supplementation cannot be excluded and may partly explain differences between studies.

Furthermore, no significant differences in antiresorptive therapy duration or AAOMS stage were observed between mandibular and maxillary MRONJ, indicating that these variables were not associated with lesion location in the present cohort and were therefore unlikely to explain the observed findings.

Although the observational design of the present study does not permit causal inference, several biological mechanisms may explain the observed findings. Beyond its established role in bone metabolism, vitamin D regulates the RANK/RANKL/OPG signaling pathway, supports angiogenesis, and contributes to bone remodeling and tissue repair, providing a plausible biological explanation for the observed association [33]. In patients receiving antiresorptive therapy, impaired bone turnover combined with vitamin D deficiency may further compromise the regenerative capacity of the jawbone [34]. Furthermore, because the mandible is characterized by higher cortical bone density, relatively limited vascularization, and greater biomechanical loading during mastication, these local anatomical features may increase its susceptibility to impaired healing when systemic factors such as reduced vitamin D status are present [7,35,36,37]. In addition to systemic factors, local oral conditions may also influence the development and progression of MRONJ. Poor oral hygiene and periodontal disease may contribute to chronic oral inflammation and bacterial colonization of exposed bone, potentially exacerbating local conditions associated with MRONJ. Recent observational evidence indicates that poor oral hygiene is common among patients with MRONJ and may be associated with more advanced disease; however, its direct role in MRONJ development remains uncertain [38].

Mandibular involvement predominated across all treatment groups, consistent with previous reports suggesting greater anatomical susceptibility of the mandible [3,39,40,41,42,43]. Moreover, lower serum vitamin D levels remained independently associated with mandibular MRONJ after adjustment for age, sex, diabetes mellitus, and antiresorptive therapy in multivariable logistic regression, supporting an independent association between vitamin D status and lesion location.

Another notable result of this study was the identification of a moderate positive correlation between patient age and the number of extracted teeth (*r* = 0.440, *p* < 0.001). Although this association likely reflects the cumulative burden of oral disease and dental interventions in older patients, no significant correlation was observed between age and serum vitamin D levels. Elderly patients often present chronic periodontal disease, cumulative oral pathology, and a history of multiple dentoalveolar interventions, all of which are recognized as important local risk factors for MRONJ. In addition to tooth extractions, persistent odontogenic infections, including apical periodontitis and endodontic treatment failure, have also been recognized as important local inflammatory triggers for MRONJ. Recent clinical evidence indicates that endodontic treatment failure and persistent periapical disease may act as local risk factors for MRONJ, particularly in oncologic patients treated with antiresorptive agents [44]. Because the present study evaluated only the number of extracted teeth and not the underlying dental pathology leading to extraction, the potential influence of these conditions could not be assessed and should be systematically evaluated in future prospective studies. Beyond these local factors, the age-related reduction in bone regenerative capacity and the presence of multiple systemic comorbidities may further contribute to more extensive oral involvement in these patients [19,43,45,46,47,48].

Correlation analyses were performed to explore whether serum vitamin D levels were associated with patient age or cumulative dental interventions, both of which may influence bone metabolism and susceptibility to MRONJ. No significant correlations were identified between serum vitamin D levels and either age or the number of extracted teeth, suggesting that vitamin D status in this cohort was independent of these clinical variables.

The findings of the present study emphasize the clinical importance of routine biochemical evaluation, particularly serum vitamin D assessment, in patients receiving antiresorptive therapy or undergoing invasive dental procedures. Lower serum vitamin D levels observed in patients with mandibular MRONJ lesions suggest the need for careful clinical monitoring and individualized preventive management in these patients. Preventive dental evaluation before initiating antiresorptive therapy may also help reduce the risk of MRONJ and facilitate the identification of patients who require closer follow-up.

Overall, these results support the importance of an interdisciplinary approach involving oral and maxillofacial surgeons, oncologists, and endocrinologists in the prevention and management of patients at increased risk of developing MRONJ.

Despite the clinical relevance of our findings, the study has several limitations. First, the retrospective design may introduce selection and information bias. Second, the relatively small sample size limited the statistical power and generalizability of the findings. Although lower serum vitamin D levels remained independently associated with mandibular MRONJ, the retrospective observational design precludes causal inference. Therefore, these findings should be considered hypothesis-generating and require confirmation in larger prospective studies. Another important limitation is the absence of a matched control group of antiresorptive-treated patients without MRONJ. Consequently, it was not possible to determine whether the observed vitamin D levels were specific to MRONJ or reflected characteristics of patients receiving antiresorptive therapy. Furthermore, information regarding vitamin D supplementation, fasting status, renal function, oral hygiene, baseline periodontal status, and the underlying dental pathology leading to tooth extraction was not consistently available, leaving the possibility of residual confounding. Detailed data on MRONJ treatment modalities and long-term clinical outcomes (including healing, recurrence, and disease progression) were also unavailable because of the retrospective design. In addition, the low frequency of several concomitant medications (e.g., corticosteroid therapy) precluded reliable adjustment for their potential confounding effects. Although seasonal differences in serum vitamin D concentrations were evaluated, residual confounding related to sun exposure, dietary habits, laboratory methodology, and geographic factors cannot be excluded. Future prospective multicenter studies with standardized assessment of these variables are required to better define the independent role of vitamin D in MRONJ. Nevertheless, our study provides clinically relevant and homogeneous data that can serve as a valuable basis for future multicenter research.

## 5. Conclusions

In this retrospective study, lower serum vitamin D levels were associated with mandibular MRONJ involvement, whereas serum vitamin D levels were not significantly associated with patient age, extraction history, or the type of antiresorptive therapy. These findings suggest that vitamin D status may be related to specific clinical characteristics of MRONJ; however, the observational design of the study does not allow conclusions regarding causality. Further prospective multicenter studies are required to clarify the role of vitamin D in MRONJ pathogenesis and validate its potential as a clinically useful biomarker in patients receiving antiresorptive therapy.

## Figures and Tables

**Table 1:** Baseline demographic and clinical characteristics of patients with MRONJ according to antiresorptive therapy.

Characteristic	Zoledronic Acid (*n* = 30)	Denosumab (*n* = 22)	Oral Bisphosphonates (*n* = 20)	*p*-Value
Age (years), mean ± SD	62.3 ± 9.1	66.5 ± 8.9	66.6 ± 9.0	0.157
Female sex, *n* (%)	18 (60.0)	13 (59.1)	20 (100)	**0.003**
Urban origin, *n* (%)	20 (66.7)	10 (45.5)	8 (40.0)	0.128
Smoking, *n* (%)	23 (76.7)	16 (72.7)	13 (65.0)	0.664
Serum vitamin D levels (µg/L), mean ± SD	26.3 ± 5.7	28.9 ± 7.3	24.6 ± 7.0	0.110
Number of extracted teeth, mean ± SD	1.53 ± 0.51	1.81 ± 0.87	1.75 ± 0.72	0.324
Mandibular location, *n* (%)	19 (63.3)	12 (54.5)	15 (75.0)	0.385
Diabetes mellitus, *n* (%)	9 (30.0)	8 (36.4)	8 (40.0)	0.753
Cardiovascular disease, *n* (%)	14 (46.7)	13 (59.1)	10 (50.0)	0.668
Thyroid disease, *n* (%)	0 (0)	1 (4.5)	5 (25.0)	**0.005**
Duration of antiresorptive therapy (months), mean ± SD	9.0 ± 2.5	13.7 ± 2.2	42.5 ± 12.3	**<0.001**
AAOMS stage, *n* (%)				0.988
Stage I	10 (33.3)	7 (31.8)	7 (35.0)	
Stage II	13 (43.3)	11 (50.0)	9 (45.0)	
Stage III	7 (23.3)	4 (18.2)	4 (20.0)	

SD = standard deviation; *n* = number of patients. Statistically significant *p*-values are shown in bold.

**Table 2:** Concomitant medications according to antiresorptive therapy group.

Medication	Zoledronic Acid (*n* = 30)	Denosumab (*n* = 22)	Oral Bisphosphonates (*n* = 20)	*p*-Value
Antihypertensive medication, *n* (%)	7 (23.3)	8 (36.4)	5 (25.0)	0.554
Anticoagulant therapy, *n* (%)	7 (23.3)	8 (36.3)	8 (40.0)	0.403
Antidiabetic medication, *n* (%)	9 (30.0)	8 (36.4)	8 (40.0)	0.753
Corticosteroid therapy, *n* (%)	0 (0)	0 (0)	1 (5.0)	0.268
Thyroid medication, *n* (%)	0 (0.0)	1 (4.5)	5 (25.0)	**0.005**

*n* = number of patients. Statistically significant *p*-values are shown in bold.

**Table 3:** Correlation analysis between age, serum vitamin D levels, and the number of extracted teeth.

Variables	Pearson (*r*)	*p*-Value	Spearman (*ρ)*	*p*-Value
Age vs. serum vitamin D	−0.202	0.089	−0.207	0.081
Age vs. number of extracted teeth	**0.440**	**<0.001**	**0.395**	**<0.001**
Serum vitamin D vs. number of extracted teeth	−0.204	0.088	−0.144	0.232

Pearson’s (*r*) and Spearman’s (*ρ*) correlation coefficients are presented. Statistically significant *p*-values are shown in bold.

**Table 4:** Comparison between mandibular and maxillary MRONJ cases.

Variable	Mandibular (*n* = 46)	Maxillary (*n* = 26)	*p*-Value
Age (years), mean ± SD	63.6 ± 9.8	66.8 ± 7.6	0.125
Serum vitamin D levels (µg/L), mean ± SD	25.4 ± 6.6	28.7 ± 6.5	**0.046**
Number of extracted teeth, mean ± SD	1.71 ± 0.79	1.62 ± 0.50	0.532
Smoking, *n* (%)	32 (69.6)	20 (76.9)	0.503
Diabetes mellitus, *n* (%)	18 (39.1)	7 (26.9)	0.296
Female sex, *n* (%)	31 (67.4)	20 (76.9)	0.393
Zoledronic acid, *n* (%)	19 (41.3)	11 (42.3)	0.385
Denosumab, *n* (%)	12 (26.1)	10 (38.5)	
Oral bisphosphonates, *n* (%)	15 (32.6)	5 (19.2)	

SD = standard deviation; *n* = number of patients. Statistically significant *p*-values are shown in bold.

**Table 5:** Multivariable binary logistic regression analysis of factors associated with mandibular MRONJ.

Variable	OR	95% CI	*p*-Value
Serum vitamin D (µg/L)	0.912	0.834–0.998	**0.044**
Age (years)	0.928	0.867–0.992	**0.028**
Male sex	2.541	0.698–9.247	0.157
Diabetes mellitus	1.712	0.526–5.570	0.371
Denosumab vs. zoledronic acid	1.086	0.301–3.914	0.900
Oral bisphosphonates vs. zoledronic acid	2.954	0.674–12.938	0.151

OR = odds ratio; CI = confidence interval. Outcome: mandibular MRONJ. Reference categories: female sex, no diabetes mellitus, and zoledronic acid therapy. Statistically significant *p*-values are shown in bold.

## Data Availability

The data presented in this study are available on request from the corresponding author.

## Abbreviations

The following abbreviations are used in this manuscript:

MRONJ	Medication-related osteonecrosis of the jaw
SD	Standard deviation
OR	Odds ratio
CI	Confidence interval
